# Crystal structures of eight- and ten-membered cyclic bis­anisyl­phosphono­thioyl disulfanes and comparison with their *P*-ferrocenyl analogues

**DOI:** 10.1107/S2056989018001068

**Published:** 2018-01-26

**Authors:** Witold Przychodzeń, Jarosław Chojnacki

**Affiliations:** a Department of Organic Chemistry, Gdańsk University of Technology, G.Narutowicza 11/12, 80233-PL, Gdańsk, Poland; b Department of Inorganic Chemistry, Gdańsk University of Technology, G.Narutowicza 11/12, 80233-PL, Gdańsk, Poland

**Keywords:** crystal structure, medium-sized heterocycles, bis­phosphono­thioyl disulfanes, C—H⋯S=P inter­actions

## Abstract

Two new crystal structures of eight- and ten-membered cyclic bis­anisyl­phosphono­thioyl disulfanes, namely 2,5-bis­(4-meth­oxy­phen­yl)-1,6,3,4,2λ^5^,5λ^5^-dioxadi­thiadi­phospho­cane-2,5-di­thione, C_16_H_18_O_4_P_2_S_4_, and 2,5-bis­(4-meth­oxy­phen­yl)-1,6,3,4,2λ^5^,5λ^5^-dioxadi­thia­diphosphecane-2,5-di­thione, C_18_H_22_O_4_P_2_S_4_, have been determined and compared to structures of the ferrocenyl analogues. The eight-membered rings have similar conformations (TBC) but the ten-membered macrocycles are differently puckered. Structural parameters of the relevant SPSSPS motif have been analysed and are discussed in detail. Compound **1** was refined as an inversion twin and **2** was refined as a two-component rotational twin.

## Abstract 2

Two new crystal structures of eight- and ten-membered cyclic bis­anisyl­phosphono­thioyl disulfanes have been determined and these are compared to the structures of their ferrocenyl analogues.

## Chemical context   

The most widely used sulfur-transfer agents for thio­nation of carbonyl compounds are the four-membered 2,4-dianisyl-1,3-di­thia­diphosphetane di­sulfide dimer [AnP(μ-S)S]_2_ and the 2,4-diferrocenyl-1,3-di­thia­diphosphetane di­sulfide dimer [FcP(μ-S)S]_2_, *i.e.* Lawesson reagent LR (Jesberger *et al.*, 2003 ▸) and ferrocenyl Lawesson reagent fLR (Foreman *et al.*, 1996 ▸). However, thio­phosphine oxides (AnPSO or FcPSO) separating as cyclic trimers during thio­nation reactions are usually unwanted side-products. On the other hand, the corresponding alk­oxy­phosphinodi­thioic acids, *i.e.* An(*R*O)P(S)SH and Fc(*R*O)P(S)SH, obtained in a simple reaction between LR or fLR and alcohols, are of considerable inter­est because they form a plethora of structurally inter­esting chelate complexes with metal ions (van Zyl & Woollins, 2013 ▸).

The reactions between Lawesson’s reagent and diols/diphenols have been successfully involved in the preparation of bis­(anisyl­phosphono­dithioic) acid derivatives and among them the unique eight-, nine- and ten-membered cyclic bis­anisyl­phosphono­thioyl disulfanes (Przychodzeń, 2004 ▸). A high-yielding formation of these medium-sized cyclic disulf­anes upon oxidation of bis­(anisyl­phosphono­dithtioic) acid salts by iodine proceeding without oligomeric by-products may be attributed to their fixed structure, containing the most preferred a zigzag motif of the SPSSPS unit. Slightly modified procedures with respect to the original method have recently been applied for the synthesis of related cyclic bis­(ferro­cenyl­phosphono­thio­yl)disulfanes, *e.g*. eight-membered **1** ***a*** (Pillay *et al.*, 2015 ▸) and ten-membered **2** ***a*** (Hua *et al.*, 2017 ▸) and their crystal structures have been determined. Here we report crystal structures compounds **1** and **2**, containing anisyl groups instead of the ferrocenyl moiety.
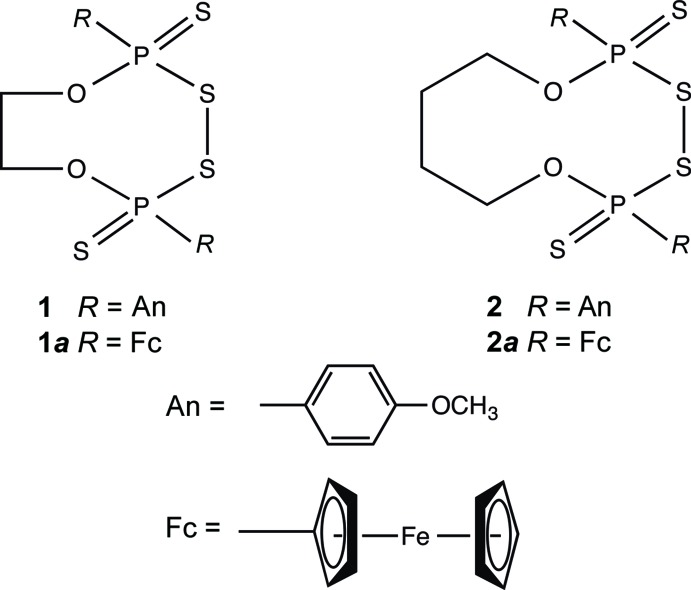


## Structural commentary   

**Figure 1:**
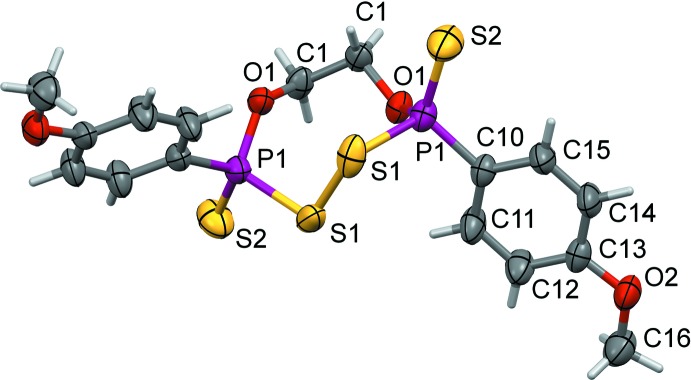
The mol­ecular structure of **1**, showing the atom-labelling scheme. Displacement ellipsoids are drawn at the 50% probability level. Symmetry-equivalent atoms are generated by the operation (*y* + 1, *x* − 1, −*z*).

Views of mol­ecular structures and atom-labeling scheme for **1** and **2** are given in Figs. 1 ▸ and 2 ▸, respectively. Compound **1** crystallizes in the *P*4_3_2_1_2 space group with a half-mol­ecule in the asymmetric unit. It follows that the mol­ecule obeys point group symmetry described by Schoenflies symbol *C*_2_ (or symbol 2 in inter­national notation). The related ferrocenyl compound **1** ***a*** crystallizes in space group *C*2/*c* with non-typical three and half independent mol­ecules in the asymmetric unit (*Z* = 28), which complicates comparisons.

Compound **2** forms a monoclinic crystalline phase obeying *P*2_1_/*c* space-group symmetry with one mol­ecule in the asymmetric unit and *Z* = 4. The related ferrocenyl structure **2** ***a*** crystallizes in space group *P* 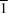 with one mol­ecule in the asymmetric unit.

The anisyl groups as well as the ferrocenyl groups on the two phospho­rus atoms are positioned in a *trans* arrangement, *i.e.* above and below the macrocycle ring plane for all compounds **1**–**2** ***a***, which is also typical for all open-chain bis­phospho­rothioyl disulfanes studied previously (Gray *et al.*, 2004 ▸).

The S—S bond lengths have values of 2.068 (2) Å for **1**; 2.0697 (10), 2.0704 (10), 2.0685 (10), 2.0711 (15) Å for **1** ***a***; 2.074 (3) Å for **2** and 2.0788 (9) Å for **2** ***a***. They are longer than the typical S—S bond lengths for known diorganyl disulfanes *R*SS*R* [2.05 (3) Å]. The observed S—S bond elongation in **1**–**2** ***a*** may be correlated with the PSSP torsion angles (Knopik *et al.*, 1993 ▸). As expected, exocyclic P=S bond lengths (*ca* 1.92 Å) are shorter than the endocyclic P—S bonds (*ca* 2.10 Å).

All phospho­rus atoms in **1**–**2** ***a*** adopt a distorted tetra­hedral geometry, where the C—P=S angles deviated the most (116.1–118.5°) from the ideal tetra­hedral angle. This is obviously due to the steric effects of the anisyl and ferrocenyl substituents. On the other hand, it is worthy to note that the O—P–S bond angles in **1**–**2** ***a*** (107–108°) are not distorted, probably due to minimal conformational strain present in those medium-sized heterocycles. Moreover, both the P=S and aromatic anisyl groups in **1** are almost perfectly coplanar (unlike P=S and the cyclopentadienyl ring in **1** ***a***), which provides energetically favorable conjugation [torsion angle S2—P1—C10—C15 = −3.8 (4)° in **1** *vs* 35.75 (3)° for the equivalent angle in a selected representative mol­ecule with Fe7 in **1** ***a***]. The other related independent torsion angles in **1** ***a*** are −31. (3), −33.9 (3), −27.0 (3), −28.7 (3), 34.8 (3), 35.7 (3)°, for Fe1–Fe6, respectively.

It is well recognised that PSSP torsion is a characteristic feature of all disulfanes as a class of organic compounds. The structure of **1** is the most symmetric with the lowest PSSP torsion [−93.68 (8)°] and shows only a moderate deviation from a right angle. The PSSP torsion angles in **1** ***a*** [−101.19 (4), −100.06 (4), −101.47 (4) and 99.89 (4)°] are 6–8° wider than in **1**. Notably, ten-membered disulfanes have even wider PSSP torsion angles and the difference between them is smaller, −112.89 (11) and 114.9 (4)°, for **2** and **2** ***a***, respectively.

Only non-classical hydrogen-bonding inter­actions of the type C—H⋯*X* (*X* = O or S) can be found in the structures of **1** and **2** (Tables 1 ▸ and 2 ▸).

The transannular P⋯P distances are very similar within the same ring size and increase, from 4.3331 (17) Å in **1** and 4.2625 (9), 4.2670 (9), 4.2652 (9) or 4.261 (1) Å (for different independent mol­ecules in **1** ***a***) for eight-membered rings, to 4.614 (2) in **2** and 4.604 (1) Å in **2** ***a*** for the ten-membered rings.

**Figure 3:**
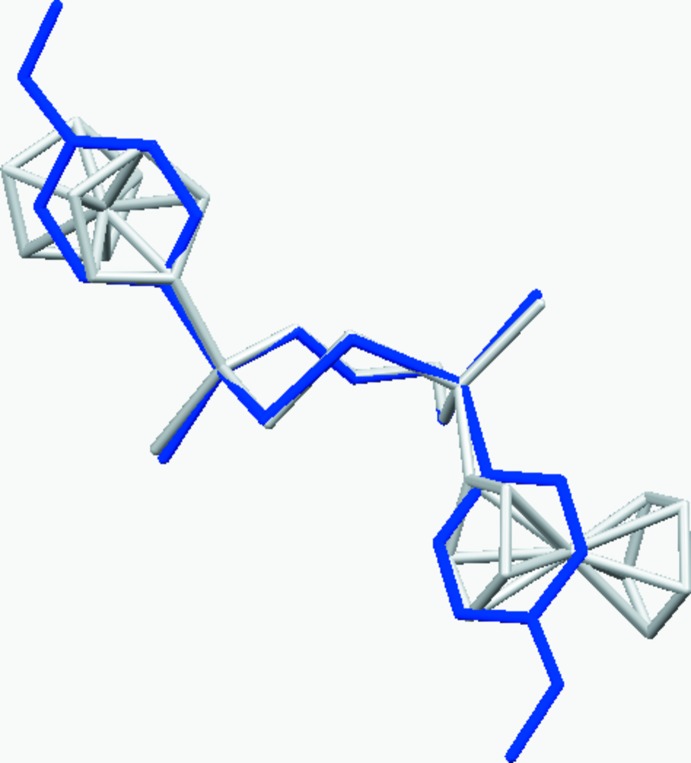
Superimposition of eight-membered di­phospho­canes **1** (blue) and **1** ***a*** (grey) based on the best PSSP fragment fit.

**Figure 4:**
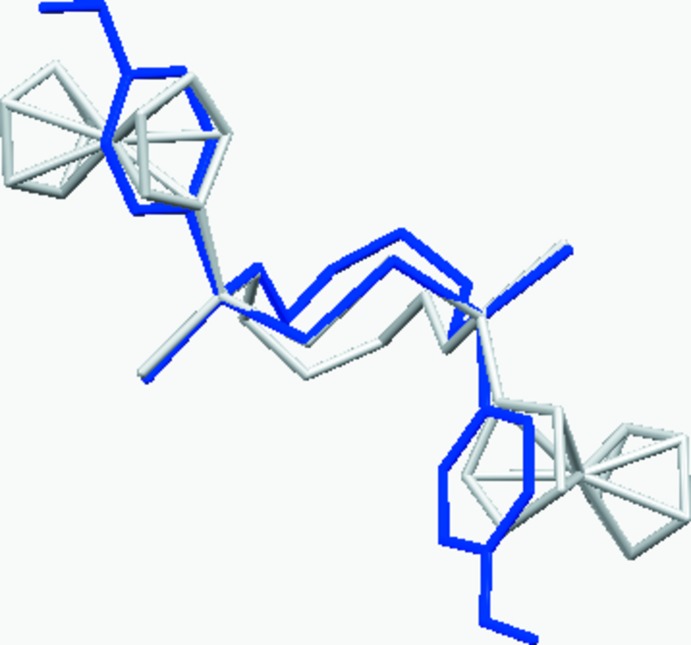
Overlay of ten-membered diphosphecanes **2** (blue) and **2** ***a*** (grey) based on the best PSSP fragment fit.

The conformation of the eight-membered macrocycles in **1** and **1** ***a*** was recognised by *PLATON* (Spek, 2009 ▸) as being closest to the TBC form (twist–boat chair; Evans & Boeyens, 1989 ▸; Wiberg, 2003 ▸), which is consistent with *C*_2_ point symmetry. Fig. 3 ▸ shows the overlay of the two structures based on the best fit of the PSSP fragment. The conformation of **2** was not assigned to any border type by *PLATON*, but Fig. 4 ▸ shows the puckering in **2** and **2** ***a*** is distinctively different.

It is probably important to note that the intra­molecular C4—H4*B*⋯O1 hydrogen bond (Table 2 ▸) stabilizes the ten-membered ring of **2**.

## Supra­molecular features   

The strongest inter­molecular hydrogen-bonding inter­action in **1** is between the anisyl *ortho*-hydrogen and macrocyclic O1 atoms and links the mol­ecules into a diamondoid network. There are no ring-stacking inter­actions since the shortest centroid–centroid distance is 5.0965 (3) Å. The anisyl substituents may have inhibited this kind of inter­action.

**Figure 5:**
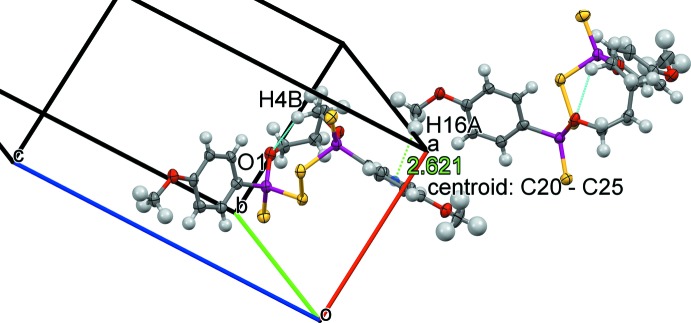
C—H⋯π inter­action and inter­nal C—H⋯O hydrogen bonding in the ten-membered ring of **2**.

Inter­molecular inter­actions in **2** are mainly based on the anisyl methoxyl CH_3_O oxygen atoms O3 and O4 and the P=S sulfur atom S3 as acceptors. Hydrogen-bond donors are the anisyl *ortho*-hydrogen atoms or methyl­ene hydrogen atoms. Moreover, some C—H..π. inter­actions may play some role in the system, *e.g*. C16—H16*A*⋯ring(C20–C25), see Fig. 5 ▸. Again, the stacking inter­actions are weak since the closest inter­centroid distance is equal to 4.9213 (4) Å.

## Database survey   

Bisphosphono­thioyl disulfanes represent a rather rare class of compounds (CSD Version 5.28, updated to Nov. 2016; Groom *et al.*, 2016 ▸). Only three structures of cyclic bis­phosphono­thioyl disulfanes can be found in the database, HUGXAK, HUXEO and HUGXIS (ferrocenyl derivatives; Pillay *et al.*, 2015 ▸) and four more will be available there soon (Hua *et al.*, 2017 ▸). For structures of acyclic bis­phosphono­thioyl disulfanes see: FATTEA, FATTIE, FATVEC (Gray *et al.*, 2004 ▸), YESDIY (Łopusiński *et al.*, 1991 ▸), SIZHUF (Przychodzeń & Chojnacki, 2008 ▸) and WAYMEO (Knopik *et al.*, 1993 ▸).

## Synthesis and crystallization   

Eight- and ten-membered cyclic bis­anisyl­phosphono­thioyl disulfanes **1** and **2** were prepared using previously reported procedure (Przychodzeń, 2004 ▸). Compound **1** was fully spectroscopically characterized in that paper. Disulfane **2** is quite new, so all available spectroscopic data are given below. Both **1** and **2** gave good quality colourless crystals after crystallization from ethyl acetate–cyclo­hexane (1:2 *v*/*v*) solvent system.

**2,5-Bis(4-meth­oxy­phen­yl)-1,6,3,4,2,5-dioxadi­thiadi­phos­pho­cane 2,5-di­thione, 1**

M.p. 441-443 K.

**2,5-Bis(4-meth­oxy­phen­yl)-1,6,3,4,2,5-dioxadi­thia­diphos­phecane 2,5-di­thione, 2**

Yield: 65%, m.p. 415–417 K.

^1^H NMR (CDCl_3_): 2.20 (*m*, 2H, OCH_2_C*H_2_*), 2.25 (*m*, 2H, OCH_2_C*H_2_*), 3.89 (*s*, 6H, OC*H_3_*), 4.37 (*dddd*, ^3^ *J*_HH_ = 11.6 Hz, ^2^ *J*_HH_ = 10.4 Hz, ^3^ *J*_HP_ = 5.4 Hz, ^3^ *J*_HH_ = 2.2 Hz, 2H, OC*H_A_*H_B_), 4.89 (*ddt*, ^2^ *J*_HH_ = 10.4 Hz, ^3^ *J*_HP_ = 9.3 Hz, ^3^ *J*_HH_ = 3.5 Hz, 2H, OCH_A_ *H_B_*), 7.01 (*dd*, ^3^ *J*_HH_ = 8.8 Hz, ^4^ *J*_HP_ = 3.9 Hz, 4H, H_meta_), 7.87 (*dd*, ^3^ *J*_HP_ = 14.2 Hz, ^3^ *J*_HH_ = 8.8 Hz, 4H, H_ortho_).

^13^C NMR: 27.21 (*d*, *J* = 6.9 Hz), 55.46 (*s*), 67.08 (*d*, *J* = 6.3 Hz), 114.03 (*d*, *J* = 17.5 Hz), 125.41 (*d*, *J* = 134 Hz), 132.89 (*d*, *J* = 14.5 Hz), 163.09 (*s*).

^31^P{^1^H} NMR (CDCl_3_): 89.19 (^3^ *J*_PP_ = 4 Hz)

MS calculated for C_18_H_22_O_4_P_2_S_4_: 492.0. Found: 492.9 [*M*+H]^+^.

## Refinement   

Crystal data, data collection and structure refinement details are summarized in Table 3 ▸. Structure **1** was refined as an inversion twin with contribution of the second domain equal to 0.45 (17). This explains the ambiguous Flack parameter and is not surprising since we started from achiral substrates. Structure **2** was refined as a two-component rotational twin with twin law: {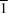 0 0, 0 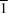 0, 0 0 1} and BASF = 0.767 (3). Relatively high residual electron-density peaks in **2** (*Q*1–*Q*3 *ca* 2e Å^3^), which are close to sulfur atoms (0.58 Å from S4, 0.49 Å from S2, 0.49 Å from S1), may stem from conformational flexibility of the ring. Note: the structure of **1** was determined at room temperature (due to a failure of our CryoStream unit) not at 120 K as for **2** but we believe it did not influence the qualitative conclusions drawn from the results.

## Supplementary Material

Crystal structure: contains datablock(s) 2, global, 1. DOI: 10.1107/S2056989018001068/zp2026sup1.cif

Structure factors: contains datablock(s) 1. DOI: 10.1107/S2056989018001068/zp20261sup2.hkl

Structure factors: contains datablock(s) 2. DOI: 10.1107/S2056989018001068/zp20262sup3.hkl

Supporting information file. DOI: 10.1107/S2056989018001068/zp20261sup4.cml

Supporting information file. DOI: 10.1107/S2056989018001068/zp20262sup5.cml

CCDC references: 1558043, 719124

Additional supporting information:  crystallographic information; 3D view; checkCIF report

## Figures and Tables

**Figure 2:**
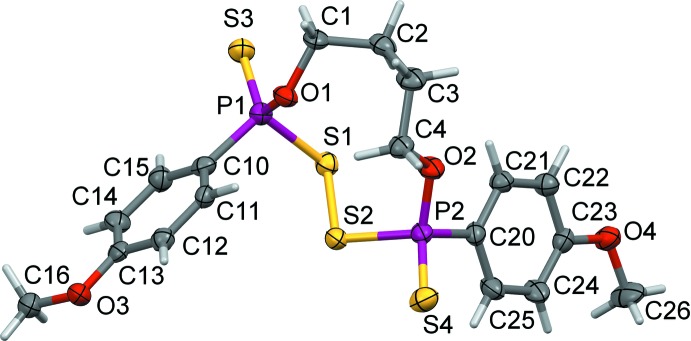
View of the asymmetric unit of **2**, showing the atom-labelling scheme. Displacement ellipsoids are drawn at the 50% probability level.

**Table 1:** Hydrogen-bond geometry (Å, °) for **1** [Chem scheme1]

*D*—H⋯*A*	*D*—H	H⋯*A*	*D*⋯*A*	*D*—H⋯*A*
C1—H1*A*⋯O2^i^	0.97	2.60	3.4843 (2)	151
C14—H14⋯O1^ii^	0.93	2.55	3.4548 (2)	163

Symmetry codes: (i) 
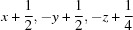
; (ii) 
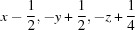
.

**Table 2:** Hydrogen-bond geometry (Å, °) for **2** [Chem scheme1] *Cg* is the centroid of the C20–C25 ring.

*D*—H⋯*A*	*D*—H	H⋯*A*	*D*⋯*A*	*D*—H⋯*A*
C1—H1*B*⋯S3	0.99	2.81	3.3883 (2)	118
C4—H4*B*⋯O1	0.99	2.48	3.1308 (2)	123
C4—H4*B*⋯O4^i^	0.99	2.56	3.2708 (2)	128
C11—H11⋯O4^i^	0.95	2.62	3.4951 (3)	154
C24—H24⋯O3^ii^	0.95	2.51	3.4240 (3)	162
C16—H16*A*⋯*Cg* ^iii^	0.98	2.62	3.454 (8)	143

Symmetry codes: (i) 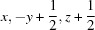; (ii) 
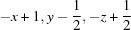
; (iii) 
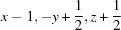
.

**Table 3:** Experimental details

	**1**	**2**
Crystal data		
Chemical formula	C_16_H_18_O_4_P_2_S_4_	C_18_H_22_O_4_P_2_S_4_
*M*_r_	464.48	492.53
Crystal system, space group	Tetragonal, *P*4_3_2_1_2	Monoclinic, *P*2_1_/*c*
Temperature (K)	296	120
*a*, *b*, *c* (Å)	7.2415 (3), 7.2415 (3), 39.516 (2)	9.4262 (6), 13.3761 (8), 17.7998 (13)
α, β, γ (°)	90, 90, 90	90, 90.068 (7), 90
*V* (Å^3^)	2072.2 (2)	2244.3 (3)
*Z*	4	4
Radiation type	Mo *K*α	Mo *K*α
μ (mm^−1^)	0.63	0.59
Crystal size (mm)	0.44 × 0.42 × 0.03	0.21 × 0.20 × 0.14

Data collection		
Diffractometer	Oxford Diffraction KM-4 CCD	Oxford Diffraction KM-4 CCD
Absorption correction	Multi-scan (*CrysAlis PRO*; Agilent, 2011 ▸)	Analytical [*CrysAlis PRO* (Agilent, 2011 ▸) based on expressions derived by Clark & Reid (1995 ▸)]
*T*_min_, *T*_max_	0.689, 0.98	0.893, 0.929
No. of measured, independent and observed [*I* > 2σ(*I*)] reflections	14211, 2019, 1839	9496, 4047, 3309
*R*_int_	0.036	0.051
(sin θ/λ)_max_ (Å^−1^)	0.617	0.606

Refinement		
*R*[*F* ^2^ > 2σ(*F* ^2^)], *wR*(*F* ^2^), *S*	0.038, 0.092, 1.09	0.082, 0.241, 1.05
No. of reflections	2019	4047
No. of parameters	120	256
H-atom treatment	H-atom parameters constrained	H-atom parameters constrained
Δρ_max_, Δρ_min_ (e Å^−3^)	0.33, −0.22	2.27, −0.84
Absolute structure	Refined as an inversion twin	–
Absolute structure parameter	0.45 (17)	–

Computer programs: *CrysAlis PRO* (Agilent, 2011 ▸), *SHELXT* (Sheldrick, 2015*a* ▸), *SHELXL2014* (Sheldrick, 2015*b* ▸), *Mercury* (Macrae *et al.*, 2008 ▸), *WinGX* (Farrugia, 2012 ▸) and *publCIF* (Westrip, 2010 ▸).

## supplementary crystallographic information

### 2,5-Bis(4-methoxyphenyl)-1,6,3,4,2λ5,5λ5-dioxadithiadiphosphocane-2,5-dithione (1) Crystal data

**Table:**

C_16_H_18_O_4_P_2_S_4_	*D*_x_ = 1.489 Mg m^−^^3^
*M**_r_* = 464.48	Mo *K*α radiation, λ = 0.71073 Å
Tetragonal, *P*4_3_2_1_2	Cell parameters from 6759 reflections
Hall symbol: P 4nw 2abw	θ = 2.1–32.4°
*a* = 7.2415 (3) Å	µ = 0.63 mm^−^^1^
*c* = 39.516 (2) Å	*T* = 296 K
*V* = 2072.2 (2) Å^3^	Plate, colourless
*Z* = 4	0.44 × 0.42 × 0.03 mm
*F*(000) = 960	

### 2,5-Bis(4-methoxyphenyl)-1,6,3,4,2λ5,5λ5-dioxadithiadiphosphocane-2,5-dithione (1) Data collection

**Table:**

Oxford Diffraction KM-4 CCD diffractometer	1839 reflections with *I* > 2σ(*I*)
Detector resolution: 8.1883 pixels mm^-1^	*R*_int_ = 0.036
ω scans, 0.40 deg width	θ_max_ = 26.0°, θ_min_ = 2.9°
Absorption correction: multi-scan (CrysAlis PRO; Agilent, 2011)	*h* = −8→8
*T*_min_ = 0.689, *T*_max_ = 0.98	*k* = −8→8
14211 measured reflections	*l* = −48→38
2019 independent reflections	

### 2,5-Bis(4-methoxyphenyl)-1,6,3,4,2λ5,5λ5-dioxadithiadiphosphocane-2,5-dithione (1) Refinement

**Table:**

Refinement on *F*^2^	Secondary atom site location: difference Fourier map
Least-squares matrix: full	Hydrogen site location: inferred from neighbouring sites
*R*[*F*^2^ > 2σ(*F*^2^)] = 0.038	H-atom parameters constrained
*wR*(*F*^2^) = 0.092	*w* = 1/[σ^2^(*F*_o_^2^) + (0.0514*P*)^2^ + 0.5163*P*] where *P* = (*F*_o_^2^ + 2*F*_c_^2^)/3
*S* = 1.09	(Δ/σ)_max_ < 0.001
2019 reflections	Δρ_max_ = 0.33 e Å^−^^3^
120 parameters	Δρ_min_ = −0.22 e Å^−^^3^
0 restraints	Absolute structure: Refined as an inversion twin
Primary atom site location: structure-invariant direct methods	Absolute structure parameter: 0.45 (17)

### 2,5-Bis(4-methoxyphenyl)-1,6,3,4,2λ5,5λ5-dioxadithiadiphosphocane-2,5-dithione (1) Special details

**Table:**

Geometry. All esds (except the esd in the dihedral angle between two l.s. planes) are estimated using the full covariance matrix. The cell esds are taken into account individually in the estimation of esds in distances, angles and torsion angles; correlations between esds in cell parameters are only used when they are defined by crystal symmetry. An approximate (isotropic) treatment of cell esds is used for estimating esds involving l.s. planes.
Refinement. Refined as a 2-component inversion twin.

### 2,5-Bis(4-methoxyphenyl)-1,6,3,4,2λ5,5λ5-dioxadithiadiphosphocane-2,5-dithione (1) Fractional atomic coordinates and isotropic or equivalent isotropic displacement parameters (Å^2^)

**Table:**

	*x*	*y*	*z*	*U*_iso_*/*U*_eq_	
P1	0.93887 (12)	0.21832 (13)	0.04117 (2)	0.0424 (2)	
S1	0.84270 (13)	0.04364 (17)	0.00256 (2)	0.0584 (3)	
S2	0.79007 (17)	0.43697 (17)	0.03709 (3)	0.0715 (4)	
O1	1.1556 (3)	0.2344 (3)	0.03669 (5)	0.0424 (6)	
O2	0.8974 (4)	−0.1523 (4)	0.17469 (6)	0.0552 (7)	
C1	1.2424 (5)	0.3553 (5)	0.01202 (8)	0.0450 (8)	
H1A	1.3209	0.4439	0.0235	0.054*	
H1B	1.1485	0.4228	−0.0004	0.054*	
C10	0.9277 (5)	0.1007 (5)	0.08087 (8)	0.0412 (7)	
C11	1.0238 (5)	−0.0610 (6)	0.08670 (9)	0.0576 (10)	
H11	1.0949	−0.1113	0.0694	0.069*	
C12	1.0170 (6)	−0.1496 (6)	0.11749 (9)	0.0584 (10)	
H12	1.082	−0.2589	0.1207	0.07*	
C13	0.9144 (5)	−0.0768 (5)	0.14352 (7)	0.0434 (8)	
C14	0.8206 (6)	0.0864 (6)	0.13813 (9)	0.0555 (10)	
H14	0.7529	0.1384	0.1557	0.067*	
C15	0.8252 (6)	0.1736 (5)	0.10720 (9)	0.0526 (9)	
H15	0.759	0.2823	0.104	0.063*	
C16	1.0028 (6)	−0.3130 (6)	0.18232 (11)	0.0672 (12)	
H16A	0.9613	−0.4138	0.1685	0.101*	
H16B	0.9871	−0.3442	0.2058	0.101*	
H16C	1.1309	−0.2894	0.1779	0.101*	

### 2,5-Bis(4-methoxyphenyl)-1,6,3,4,2λ5,5λ5-dioxadithiadiphosphocane-2,5-dithione (1) Atomic displacement parameters (Å^2^)

**Table:**

	*U*^11^	*U*^22^	*U*^33^	*U*^12^	*U*^13^	*U*^23^
P1	0.0373 (4)	0.0559 (5)	0.0340 (4)	0.0025 (4)	0.0018 (3)	0.0025 (4)
S1	0.0423 (5)	0.0932 (8)	0.0396 (5)	−0.0164 (5)	−0.0034 (4)	−0.0029 (5)
S2	0.0711 (7)	0.0755 (7)	0.0678 (7)	0.0285 (6)	0.0106 (5)	0.0156 (6)
O1	0.0403 (12)	0.0552 (14)	0.0318 (11)	−0.0057 (11)	−0.0003 (9)	0.0010 (10)
O2	0.0598 (16)	0.0694 (17)	0.0363 (13)	0.0070 (13)	0.0086 (11)	0.0069 (11)
C1	0.054 (2)	0.0427 (18)	0.0381 (17)	−0.0144 (16)	0.0004 (15)	−0.0036 (14)
C10	0.0358 (16)	0.054 (2)	0.0336 (15)	0.0004 (15)	0.0034 (14)	0.0006 (14)
C11	0.056 (2)	0.076 (3)	0.0400 (18)	0.026 (2)	0.0145 (16)	0.0035 (18)
C12	0.062 (2)	0.069 (2)	0.0443 (19)	0.0260 (19)	0.0073 (17)	0.0058 (17)
C13	0.0363 (18)	0.061 (2)	0.0329 (15)	−0.0035 (15)	0.0030 (13)	0.0010 (15)
C14	0.066 (3)	0.061 (2)	0.0402 (18)	0.0157 (19)	0.0169 (16)	−0.0040 (17)
C15	0.065 (3)	0.050 (2)	0.0424 (19)	0.0145 (18)	0.0131 (17)	−0.0021 (16)
C16	0.073 (3)	0.078 (3)	0.051 (2)	0.011 (2)	0.0064 (19)	0.016 (2)

### 2,5-Bis(4-methoxyphenyl)-1,6,3,4,2λ5,5λ5-dioxadithiadiphosphocane-2,5-dithione (1) Geometric parameters (Å, º)

**Table:**

P1—O1	1.584 (2)	C10—C15	1.383 (5)
P1—C10	1.787 (3)	C11—C12	1.376 (5)
P1—S2	1.9220 (14)	C11—H11	0.93
P1—S1	2.1006 (13)	C12—C13	1.374 (5)
S1—S1^i^	2.068 (2)	C12—H12	0.93
O1—C1	1.453 (4)	C13—C14	1.380 (5)
O2—C13	1.353 (4)	C14—C15	1.376 (5)
O2—C16	1.424 (5)	C14—H14	0.93
C1—C1^i^	1.496 (7)	C15—H15	0.93
C1—H1A	0.97	C16—H16A	0.96
C1—H1B	0.97	C16—H16B	0.96
C10—C11	1.382 (5)	C16—H16C	0.96
			
O1—P1—C10	100.26 (14)	C10—C11—H11	119.2
O1—P1—S2	119.05 (11)	C13—C12—C11	120.2 (4)
C10—P1—S2	116.15 (13)	C13—C12—H12	119.9
O1—P1—S1	106.97 (9)	C11—C12—H12	119.9
C10—P1—S1	109.61 (12)	O2—C13—C12	125.1 (3)
S2—P1—S1	104.44 (6)	O2—C13—C14	116.2 (3)
S1^i^—S1—P1	105.17 (6)	C12—C13—C14	118.6 (3)
C1—O1—P1	123.2 (2)	C15—C14—C13	121.2 (3)
C13—O2—C16	118.3 (3)	C15—C14—H14	119.4
O1—C1—C1^i^	109.5 (3)	C13—C14—H14	119.4
O1—C1—H1A	109.8	C14—C15—C10	120.4 (3)
C1^i^—C1—H1A	109.8	C14—C15—H15	119.8
O1—C1—H1B	109.8	C10—C15—H15	119.8
C1^i^—C1—H1B	109.8	O2—C16—H16A	109.5
H1A—C1—H1B	108.2	O2—C16—H16B	109.5
C11—C10—C15	117.9 (3)	H16A—C16—H16B	109.5
C11—C10—P1	121.8 (3)	O2—C16—H16C	109.5
C15—C10—P1	120.2 (3)	H16A—C16—H16C	109.5
C12—C11—C10	121.6 (3)	H16B—C16—H16C	109.5
C12—C11—H11	119.2		
			
C10—P1—O1—C1	−165.8 (2)	P1—C10—C11—C12	179.0 (3)
S2—P1—O1—C1	−38.0 (3)	C10—C11—C12—C13	−0.6 (7)
S1—P1—O1—C1	79.8 (2)	C16—O2—C13—C12	4.4 (6)
P1—O1—C1—C1^i^	−119.6 (3)	C16—O2—C13—C14	−175.3 (4)
O1—P1—C10—C11	−52.2 (3)	C11—C12—C13—O2	179.8 (4)
S2—P1—C10—C11	178.1 (3)	C11—C12—C13—C14	−0.5 (6)
S1—P1—C10—C11	60.1 (3)	O2—C13—C14—C15	−178.8 (4)
O1—P1—C10—C15	125.9 (3)	C12—C13—C14—C15	1.5 (6)
S2—P1—C10—C15	−3.8 (4)	C13—C14—C15—C10	−1.2 (7)
S1—P1—C10—C15	−121.9 (3)	C11—C10—C15—C14	0.1 (6)
C15—C10—C11—C12	0.9 (6)	P1—C10—C15—C14	−178.1 (3)

Symmetry code: (i) *y*+1, *x*−1, −*z*.

### 2,5-Bis(4-methoxyphenyl)-1,6,3,4,2λ5,5λ5-dioxadithiadiphosphocane-2,5-dithione (1) Hydrogen-bond geometry (Å, º)

**Table:**

*D*—H···*A*	*D*—H	H···*A*	*D*···*A*	*D*—H···*A*
C1—H1*A*···O2^ii^	0.97	2.60	3.4843 (2)	151
C14—H14···O1^iii^	0.93	2.55	3.4548 (2)	163

Symmetry codes: (ii) *x*+1/2, −*y*+1/2, −*z*+1/4; (iii) *x*−1/2, −*y*+1/2, −*z*+1/4.

### 2,5-Bis(4-methoxyphenyl)-1,6,3,4,2λ5,5λ5-dioxadithiadiphosphecane-2,5-dithione (2) Crystal data

**Table:**

C_18_H_22_O_4_P_2_S_4_	*F*(000) = 1024
*M**_r_* = 492.53	*D*_x_ = 1.458 Mg m^−^^3^
Monoclinic, *P*2_1_/*c*	Mo *K*α radiation, λ = 0.71073 Å
Hall symbol: -P 2ybc	Cell parameters from 5521 reflections
*a* = 9.4262 (6) Å	θ = 1.9–28.8°
*b* = 13.3761 (8) Å	µ = 0.59 mm^−^^1^
*c* = 17.7998 (13) Å	*T* = 120 K
β = 90.068 (7)°	Prism, colourless
*V* = 2244.3 (3) Å^3^	0.21 × 0.20 × 0.14 mm
*Z* = 4	

### 2,5-Bis(4-methoxyphenyl)-1,6,3,4,2λ5,5λ5-dioxadithiadiphosphecane-2,5-dithione (2) Data collection

**Table:**

Oxford Diffraction KM-4 CCD diffractometer	4047 independent reflections
Graphite monochromator	3309 reflections with *I* > 2σ(*I*)
Detector resolution: 8.19 pixels mm^-1^	*R*_int_ = 0.051
ω scans	θ_max_ = 25.5°, θ_min_ = 1.9°
Absorption correction: analytical [CrysAlis PRO (Agilent, 2011) based on expressions derived by Clark & Reid (1995)]	*h* = −6→11
*T*_min_ = 0.893, *T*_max_ = 0.929	*k* = −13→16
9496 measured reflections	*l* = −21→18

### 2,5-Bis(4-methoxyphenyl)-1,6,3,4,2λ5,5λ5-dioxadithiadiphosphecane-2,5-dithione (2) Refinement

**Table:**

Refinement on *F*^2^	Primary atom site location: structure-invariant direct methods
Least-squares matrix: full	Secondary atom site location: difference Fourier map
*R*[*F*^2^ > 2σ(*F*^2^)] = 0.082	Hydrogen site location: inferred from neighbouring sites
*wR*(*F*^2^) = 0.241	H-atom parameters constrained
*S* = 1.05	*w* = 1/[σ^2^(*F*_o_^2^) + (0.1828*P*)^2^ + 0.8642*P*] where *P* = (*F*_o_^2^ + 2*F*_c_^2^)/3
4047 reflections	(Δ/σ)_max_ < 0.001
256 parameters	Δρ_max_ = 2.27 e Å^−^^3^
0 restraints	Δρ_min_ = −0.84 e Å^−^^3^

### 2,5-Bis(4-methoxyphenyl)-1,6,3,4,2λ5,5λ5-dioxadithiadiphosphecane-2,5-dithione (2) Special details

**Table:**

Geometry. All esds (except the esd in the dihedral angle between two l.s. planes) are estimated using the full covariance matrix. The cell esds are taken into account individually in the estimation of esds in distances, angles and torsion angles; correlations between esds in cell parameters are only used when they are defined by crystal symmetry. An approximate (isotropic) treatment of cell esds is used for estimating esds involving l.s. planes.
Refinement. Refined as a 2-component twin.

### 2,5-Bis(4-methoxyphenyl)-1,6,3,4,2λ5,5λ5-dioxadithiadiphosphecane-2,5-dithione (2) Fractional atomic coordinates and isotropic or equivalent isotropic displacement parameters (Å^2^)

**Table:**

	*x*	*y*	*z*	*U*_iso_*/*U*_eq_	
P1	0.34560 (19)	0.44281 (13)	0.17369 (10)	0.0294 (4)	
P2	0.74857 (19)	0.25294 (12)	0.13780 (10)	0.0288 (4)	
S1	0.40975 (19)	0.31382 (12)	0.11377 (10)	0.0318 (4)	
S2	0.5483 (2)	0.24408 (12)	0.18767 (10)	0.0325 (4)	
S3	0.1939 (2)	0.49814 (14)	0.11372 (11)	0.0384 (5)	
S4	0.8724 (2)	0.19798 (14)	0.21394 (12)	0.0413 (5)	
O1	0.4806 (6)	0.5097 (3)	0.1892 (3)	0.0342 (11)	
O2	0.7721 (6)	0.3641 (3)	0.1101 (3)	0.0348 (11)	
O3	0.2227 (5)	0.2847 (4)	0.4774 (3)	0.0344 (11)	
O4	0.7677 (6)	0.0419 (4)	−0.1545 (3)	0.0377 (12)	
C1	0.5242 (8)	0.5920 (5)	0.1389 (4)	0.0356 (16)	
H1A	0.5618	0.6477	0.1696	0.043*	
H1B	0.4399	0.6169	0.1114	0.043*	
C2	0.6350 (8)	0.5604 (5)	0.0833 (4)	0.0357 (16)	
H2A	0.6429	0.613	0.0444	0.043*	
H2B	0.6019	0.4987	0.0581	0.043*	
C3	0.7811 (9)	0.5412 (5)	0.1152 (5)	0.0392 (17)	
H3A	0.8083	0.5991	0.1467	0.047*	
H3B	0.8493	0.5376	0.073	0.047*	
C4	0.7962 (8)	0.4456 (5)	0.1626 (4)	0.0335 (15)	
H4A	0.8923	0.441	0.1849	0.04*	
H4B	0.7252	0.4443	0.2035	0.04*	
C10	0.3054 (7)	0.3994 (5)	0.2668 (4)	0.0303 (15)	
C11	0.4091 (7)	0.3973 (5)	0.3230 (4)	0.0288 (14)	
H11	0.501	0.4233	0.3132	0.035*	
C12	0.3784 (7)	0.3576 (5)	0.3929 (4)	0.0311 (15)	
H12	0.4494	0.3549	0.4307	0.037*	
C13	0.2422 (8)	0.3216 (5)	0.4072 (4)	0.0302 (15)	
C14	0.1390 (8)	0.3237 (5)	0.3512 (4)	0.0343 (16)	
H14	0.0465	0.2985	0.3607	0.041*	
C15	0.1728 (8)	0.3631 (5)	0.2814 (4)	0.0341 (15)	
H15	0.1026	0.3649	0.2431	0.041*	
C16	0.0843 (8)	0.2559 (5)	0.4988 (5)	0.0377 (17)	
H16A	0.0202	0.3133	0.4942	0.057*	
H16B	0.0854	0.2327	0.5511	0.057*	
H16C	0.0513	0.2017	0.4661	0.057*	
C20	0.7453 (7)	0.1893 (5)	0.0491 (4)	0.0291 (14)	
C21	0.7321 (8)	0.2409 (5)	−0.0182 (4)	0.0302 (15)	
H21	0.7173	0.3112	−0.018	0.036*	
C22	0.7406 (8)	0.1894 (5)	−0.0860 (4)	0.0320 (15)	
H22	0.7333	0.2242	−0.1323	0.038*	
C23	0.7602 (7)	0.0850 (5)	−0.0852 (4)	0.0302 (14)	
C24	0.7703 (9)	0.0347 (5)	−0.0185 (4)	0.0372 (17)	
H24	0.7819	−0.0359	−0.0183	0.045*	
C25	0.7636 (9)	0.0867 (5)	0.0488 (4)	0.0368 (16)	
H25	0.7716	0.0517	0.0951	0.044*	
C26	0.7930 (13)	−0.0639 (6)	−0.1555 (5)	0.058 (3)	
H26A	0.8789	−0.0789	−0.1266	0.087*	
H26B	0.8055	−0.0863	−0.2075	0.087*	
H26C	0.7119	−0.0986	−0.1331	0.087*	

### 2,5-Bis(4-methoxyphenyl)-1,6,3,4,2λ5,5λ5-dioxadithiadiphosphecane-2,5-dithione (2) Atomic displacement parameters (Å^2^)

**Table:**

	*U*^11^	*U*^22^	*U*^33^	*U*^12^	*U*^13^	*U*^23^
P1	0.0321 (9)	0.0271 (9)	0.0289 (9)	0.0023 (7)	0.0031 (7)	0.0019 (7)
P2	0.0358 (9)	0.0216 (8)	0.0292 (10)	0.0003 (7)	0.0061 (8)	−0.0005 (6)
S1	0.0380 (9)	0.0291 (8)	0.0285 (9)	−0.0009 (7)	0.0034 (8)	−0.0020 (7)
S2	0.0407 (9)	0.0271 (8)	0.0299 (9)	0.0032 (7)	0.0086 (8)	0.0039 (7)
S3	0.0399 (9)	0.0405 (10)	0.0348 (10)	0.0079 (7)	0.0008 (8)	0.0061 (8)
S4	0.0486 (11)	0.0347 (10)	0.0404 (11)	0.0097 (8)	−0.0055 (9)	−0.0018 (8)
O1	0.047 (3)	0.025 (2)	0.030 (3)	−0.001 (2)	0.007 (2)	0.005 (2)
O2	0.051 (3)	0.021 (2)	0.033 (3)	−0.005 (2)	0.005 (2)	−0.002 (2)
O3	0.040 (3)	0.031 (2)	0.032 (3)	0.004 (2)	0.008 (2)	0.007 (2)
O4	0.051 (3)	0.030 (3)	0.032 (3)	0.008 (2)	0.000 (2)	−0.003 (2)
C1	0.043 (4)	0.021 (3)	0.043 (4)	−0.001 (3)	0.010 (3)	0.008 (3)
C2	0.047 (4)	0.026 (3)	0.034 (4)	−0.007 (3)	0.002 (3)	0.006 (3)
C3	0.052 (4)	0.023 (3)	0.042 (4)	−0.007 (3)	0.001 (4)	0.000 (3)
C4	0.040 (4)	0.025 (3)	0.035 (4)	−0.001 (3)	0.001 (3)	−0.002 (3)
C10	0.036 (3)	0.022 (3)	0.032 (4)	0.006 (3)	−0.003 (3)	0.001 (3)
C11	0.025 (3)	0.027 (3)	0.035 (4)	0.003 (2)	0.008 (3)	0.000 (3)
C12	0.033 (4)	0.029 (3)	0.031 (4)	0.003 (3)	0.001 (3)	−0.003 (3)
C13	0.040 (4)	0.020 (3)	0.030 (4)	0.008 (3)	0.007 (3)	−0.003 (3)
C14	0.038 (4)	0.027 (3)	0.038 (4)	−0.004 (3)	0.005 (3)	0.004 (3)
C15	0.034 (4)	0.037 (4)	0.032 (4)	0.003 (3)	0.002 (3)	−0.001 (3)
C16	0.042 (4)	0.033 (4)	0.038 (4)	0.006 (3)	0.010 (3)	0.001 (3)
C20	0.030 (3)	0.026 (3)	0.031 (4)	−0.002 (3)	0.009 (3)	−0.002 (3)
C21	0.037 (4)	0.024 (3)	0.030 (4)	0.001 (3)	0.001 (3)	0.000 (3)
C22	0.035 (4)	0.028 (3)	0.033 (4)	0.000 (3)	−0.001 (3)	0.001 (3)
C23	0.028 (3)	0.029 (3)	0.034 (4)	0.003 (3)	0.003 (3)	−0.005 (3)
C24	0.057 (5)	0.020 (3)	0.035 (4)	−0.005 (3)	0.009 (4)	−0.003 (3)
C25	0.051 (4)	0.029 (4)	0.031 (4)	−0.003 (3)	0.009 (3)	0.004 (3)
C26	0.106 (8)	0.030 (4)	0.037 (5)	0.024 (5)	0.000 (5)	−0.010 (3)

### 2,5-Bis(4-methoxyphenyl)-1,6,3,4,2λ5,5λ5-dioxadithiadiphosphecane-2,5-dithione (2) Geometric parameters (Å, º)

**Table:**

P1—O1	1.580 (5)	C10—C15	1.366 (10)
P1—C10	1.798 (7)	C10—C11	1.398 (9)
P1—S3	1.931 (3)	C11—C12	1.383 (10)
P1—S1	2.117 (2)	C11—H11	0.95
P2—O2	1.582 (5)	C12—C13	1.395 (10)
P2—C20	1.794 (7)	C12—H12	0.95
P2—S4	1.933 (3)	C13—C14	1.393 (10)
P2—S2	2.091 (3)	C14—C15	1.387 (10)
S1—S2	2.074 (3)	C14—H14	0.95
O1—C1	1.477 (8)	C15—H15	0.95
O2—C4	1.453 (8)	C16—H16A	0.98
O3—C13	1.357 (8)	C16—H16B	0.98
O3—C16	1.413 (9)	C16—H16C	0.98
O4—C23	1.364 (8)	C20—C25	1.383 (10)
O4—C26	1.435 (9)	C20—C21	1.389 (10)
C1—C2	1.501 (11)	C21—C22	1.391 (10)
C1—H1A	0.99	C21—H21	0.95
C1—H1B	0.99	C22—C23	1.408 (10)
C2—C3	1.511 (11)	C22—H22	0.95
C2—H2A	0.99	C23—C24	1.368 (10)
C2—H2B	0.99	C24—C25	1.388 (10)
C3—C4	1.539 (10)	C24—H24	0.95
C3—H3A	0.99	C25—H25	0.95
C3—H3B	0.99	C26—H26A	0.98
C4—H4A	0.99	C26—H26B	0.98
C4—H4B	0.99	C26—H26C	0.98
			
O1—P1—C10	101.1 (3)	C12—C11—C10	120.3 (6)
O1—P1—S3	118.4 (2)	C12—C11—H11	119.9
C10—P1—S3	118.5 (2)	C10—C11—H11	119.9
O1—P1—S1	108.6 (2)	C11—C12—C13	119.4 (6)
C10—P1—S1	105.2 (2)	C11—C12—H12	120.3
S3—P1—S1	104.22 (11)	C13—C12—H12	120.3
O2—P2—C20	100.0 (3)	O3—C13—C14	124.9 (7)
O2—P2—S4	119.4 (2)	O3—C13—C12	114.8 (6)
C20—P2—S4	116.5 (2)	C14—C13—C12	120.3 (7)
O2—P2—S2	108.2 (2)	C15—C14—C13	119.2 (7)
C20—P2—S2	109.4 (2)	C15—C14—H14	120.4
S4—P2—S2	103.04 (11)	C13—C14—H14	120.4
S2—S1—P1	103.13 (10)	C10—C15—C14	121.1 (7)
S1—S2—P2	105.87 (10)	C10—C15—H15	119.5
C1—O1—P1	122.7 (5)	C14—C15—H15	119.5
C4—O2—P2	121.7 (4)	O3—C16—H16A	109.5
C13—O3—C16	118.3 (6)	O3—C16—H16B	109.5
C23—O4—C26	115.8 (6)	H16A—C16—H16B	109.5
O1—C1—C2	112.6 (6)	O3—C16—H16C	109.5
O1—C1—H1A	109.1	H16A—C16—H16C	109.5
C2—C1—H1A	109.1	H16B—C16—H16C	109.5
O1—C1—H1B	109.1	C25—C20—C21	120.1 (7)
C2—C1—H1B	109.1	C25—C20—P2	118.2 (6)
H1A—C1—H1B	107.8	C21—C20—P2	121.7 (5)
C1—C2—C3	115.8 (6)	C20—C21—C22	119.8 (6)
C1—C2—H2A	108.3	C20—C21—H21	120.1
C3—C2—H2A	108.3	C22—C21—H21	120.1
C1—C2—H2B	108.3	C21—C22—C23	119.4 (6)
C3—C2—H2B	108.3	C21—C22—H22	120.3
H2A—C2—H2B	107.4	C23—C22—H22	120.3
C2—C3—C4	115.5 (6)	O4—C23—C24	125.0 (6)
C2—C3—H3A	108.4	O4—C23—C22	114.6 (6)
C4—C3—H3A	108.4	C24—C23—C22	120.3 (6)
C2—C3—H3B	108.4	C23—C24—C25	120.0 (6)
C4—C3—H3B	108.4	C23—C24—H24	120
H3A—C3—H3B	107.5	C25—C24—H24	120
O2—C4—C3	104.8 (6)	C20—C25—C24	120.4 (7)
O2—C4—H4A	110.8	C20—C25—H25	119.8
C3—C4—H4A	110.8	C24—C25—H25	119.8
O2—C4—H4B	110.8	O4—C26—H26A	109.5
C3—C4—H4B	110.8	O4—C26—H26B	109.5
H4A—C4—H4B	108.9	H26A—C26—H26B	109.5
C15—C10—C11	119.8 (7)	O4—C26—H26C	109.5
C15—C10—P1	118.9 (5)	H26A—C26—H26C	109.5
C11—C10—P1	121.2 (5)	H26B—C26—H26C	109.5
			
C10—P1—O1—C1	−156.0 (5)	O3—C13—C14—C15	−179.6 (6)
S3—P1—O1—C1	−24.8 (6)	C12—C13—C14—C15	−0.7 (10)
S1—P1—O1—C1	93.6 (5)	C11—C10—C15—C14	−0.2 (10)
C20—P2—O2—C4	−173.9 (5)	P1—C10—C15—C14	176.4 (5)
S4—P2—O2—C4	−45.6 (6)	C13—C14—C15—C10	0.2 (11)
S2—P2—O2—C4	71.7 (5)	O2—P2—C20—C25	163.4 (6)
P1—O1—C1—C2	−95.9 (7)	S4—P2—C20—C25	33.1 (7)
O1—C1—C2—C3	−71.3 (8)	S2—P2—C20—C25	−83.2 (6)
C1—C2—C3—C4	72.0 (9)	O2—P2—C20—C21	−13.4 (6)
P2—O2—C4—C3	−168.1 (5)	S4—P2—C20—C21	−143.7 (5)
C2—C3—C4—O2	65.1 (8)	S2—P2—C20—C21	100.0 (6)
O1—P1—C10—C15	161.1 (5)	C25—C20—C21—C22	−1.5 (11)
S3—P1—C10—C15	29.9 (6)	P2—C20—C21—C22	175.3 (6)
S1—P1—C10—C15	−86.0 (6)	C20—C21—C22—C23	1.1 (11)
O1—P1—C10—C11	−22.4 (6)	C26—O4—C23—C24	−2.2 (11)
S3—P1—C10—C11	−153.5 (5)	C26—O4—C23—C22	177.7 (8)
S1—P1—C10—C11	90.6 (5)	C21—C22—C23—O4	−179.7 (6)
C15—C10—C11—C12	0.8 (10)	C21—C22—C23—C24	0.2 (11)
P1—C10—C11—C12	−175.8 (5)	O4—C23—C24—C25	178.9 (7)
C10—C11—C12—C13	−1.3 (10)	C22—C23—C24—C25	−1.0 (11)
C16—O3—C13—C14	−7.2 (9)	C21—C20—C25—C24	0.6 (12)
C16—O3—C13—C12	173.8 (6)	P2—C20—C25—C24	−176.2 (6)
C11—C12—C13—O3	−179.7 (6)	C23—C24—C25—C20	0.6 (12)
C11—C12—C13—C14	1.3 (10)		

### 2,5-Bis(4-methoxyphenyl)-1,6,3,4,2λ5,5λ5-dioxadithiadiphosphecane-2,5-dithione (2) Hydrogen-bond geometry (Å, º)

Cg is the centroid of the C20–C25 ring.

**Table:**

*D*—H···*A*	*D*—H	H···*A*	*D*···*A*	*D*—H···*A*
C1—H1*B*···S3	0.99	2.81	3.3883 (2)	118
C4—H4*B*···O1	0.99	2.48	3.1308 (2)	123
C4—H4*B*···O4^i^	0.99	2.56	3.2708 (2)	128
C11—H11···O4^i^	0.95	2.62	3.4951 (3)	154
C24—H24···O3^ii^	0.95	2.51	3.4240 (3)	162
C16—H16*A*···*Cg*^iii^	0.98	2.62	3.454 (8)	143

Symmetry codes: (i) *x*, −*y*+1/2, *z*+1/2; (ii) −*x*+1, *y*−1/2, −*z*+1/2; (iii) *x*−1, −*y*+1/2, *z*+1/2.
